# Designing a FAIR Catalogue of Services for the Heritage Science community

**DOI:** 10.12688/openreseurope.20798.2

**Published:** 2026-02-24

**Authors:** Laura Benassi, Jana Striova, Diego Quintero Balbas

**Affiliations:** 1 National Research Council –National Institute of Optics (CNR-INO), Largo E. Fermi 6, Florence, 50125, Italy

**Keywords:** Catalogue of services, Heritage Science, Research Infrastructure, FAIR, Open Science, Data Management, E-RIHS

## Abstract

**Background:**

The European Research Infrastructure for Heritage Science (E-RIHS), recently granted with European Research Infrastructure Consortium (ERIC) legal status, aims to advance research by facilitating access to cutting-edge scientific services and tools in the domain of heritage science. One of the major challenges and achievements during its implementation phase (2022–2024, G.A. 101079148) was the creation of the Catalogue of Services (CoS)—a digital platform that helps users find, request, and manage access to both physical and digital services offered by E-RIHS partners.

**Method:**

This paper introduces the concept, design, and development of the E-RIHS CoS, emphasising how it follows FAIR (Findable, Accessible, Interoperable, Reusable) and Open Science principles. Built with a strong focus on real research needs, the platform features a flexible and scalable architecture. It includes tools like semantic search, automated workflows, and customized dashboards based on user roles. The paper also places the CoS in the broader context of similar platforms from other research infrastructures, and point out its novel features—such as a recommendation engine, multilingual support, and advanced data analytics.

**Results and Conclusions:**

Now, the E-RIHS CoS is online, providing a single access entry to E-RIHS ERIC services and making easy to find and select the most adequate scientific services based on the users’ research questions. It is a solid and forward-thinking digital tool designed to support high-quality research, foster collaboration, and make heritage science more inclusive and accessible.

## Plain language Summary

Heritage Science is an interdisciplinary domain focused on the scientific study of cultural and natural heritage, bridging the humanities and the sciences. Researchers in this field require access to scientific laboratories and services to better understand, study, interpret, analyze, and intervene on cultural or natural objects.

Many of these services are now accessible thanks to E-RIHS, the European Research Infrastructure for Heritage Science. Its online Catalogue of Services provides an easy, research-need-oriented way to explore and access these resources.

The Catalogue was developed as a standalone FAIR (Findable, Accessible, Interoperable, Reusable) platform, integrated with automated workflows and an AI-based recommendation engine designed to assist even non-expert users in identifying the most suitable services for their projects.

Created through a co-creation process, the platform has become one of the most advanced tools available to the heritage science community and continues to evolve based on user feedback.

## Introduction

Heritage Science – a broad and multidisciplinary field that focuses on the scientific study, interpretation, conservation, and preservation of cultural and natural heritage, operates across different contexts with heterogeneous domains of knowledge and stakeholders, including non-experts and the public. In the last few decades, research on both tangible and intangible heritage has gained interest worldwide, ^1, 2^ drawing on a wide range of disciplines from the humanities (art history, archaeology, history), sciences (chemistry, physics, biology), and engineering (materials science, digital tools). ^3^ Heritage Science has progressively strengthened its collaborative nature. These efforts to enhance scientific cooperation among the different specialists involved have led to the establishment of a dedicated European Research Infrastructure (E-RIHS). ^4^

E-RIHS, included in the ESFRI roadmap in 2016 under the Social Sciences and Humanities domain and which recently became an ERIC, brings together laboratories, scientific archives, and institutions across Europe and beyond, providing integrated access to resources that support heritage-related research. Like other European RIs, E-RIHS aims at providing resources and services for research and innovation. ^5^ The services include access to facilities and expertise, data, and state-of-the-art analytical instrumentation, as well as online tools for data management and processing, to support heritage research in advancing scientific discovery and enabling collaboration across disciplines and borders.

Interdisciplinarity is a persistent challenge in the heritage science domain due to the different methods and languages employed to analyse, study, interpret, and intervene in cultural and natural heritage. The E-RIHS scientific community has worked to overcome the fragmentation of different approaches by creating three integrated platforms to offer its services:

• ARCHLAB, providing access to scientific archives and collections of heritage-related data, including access to samples, datasets, or scientific reports from historical or artistic artefacts preserved in scientific archives. ^6^
• FIXLAB, offering large and medium-scale cutting-edge laboratory instrumentation, such as synchrotrons or particle accelerators, to analyse samples coming from the artefacts under investigation. ^7^
• MOLAB, enabling the use of mobile instrumentation for on-site heritage non-invasive analysis, such as the study of an artwork directly inside the museum exhibition room. ^8^

Many online catalogues of services and marketplaces created by European and non-European research infrastructures primarily focus on digital tools, data, and related services. The E-RIHS CoS represents one of the few initiatives that effectively aligns services with user needs for access to archives, laboratories, and instrumentation. By prioritizing physical access, this approach introduces additional conceptual and operational complexity in both the design and implementation of the catalogue. To complement this framework with digital resources, E-RIHS is developing DIGILAB, a dedicated platform for digital tools and data that will provide a comprehensive, FAIR-compliant knowledge base for heritage digital twins.

To ensure effective access to scientific services, it is crucial to present the relevant information in a comprehensive, structured, and harmonized manner. This approach enhances both their visibility and discoverability. Additionally, users should be able to compare similar services easily and identify those best suited to their specific research needs. Such capabilities support a unified access model across RIs, a crucial goal also in heritage science, where users (e.g., conservators, heritage professionals, and other stakeholders) play an active role in knowledge co-creation and its translating into practice. ^2^ A Catalogue of Services (CoS) is a crucial tool for achieving these goals. In brief, it can be defined as an organized portfolio (i.e., list of services), accessible to the users (i.e., external researchers accessing the RIs services). It includes information regarding the service offered, the contact persons, and the access process. ^9, 10^

The CoS acts as a showcase and a single-entry point for managing users’ interactions among the internal and external researchers (i.e., service managers and users). Given the diversity of the disciplines involved in heritage science, as well as the widely variable expertise level of the potential users, clarity, accessibility, and guidance through co-creation and interaction with experts are critical.

This article outlines the process undertaken by the E-RIHS community over the past ten years to transition from a static, email-managed catalogue to a smarter, dynamic system powered by modern digital technologies and aligned with international standards. The development of the new E-RIHS CoS builds on prior initiatives, such as the IPERION HS European project (G.A.871034), which laid the groundwork for organizing service data and provided valuable insights into the complex, real-world needs of the heritage science community. This article documents the initial phases of the E-RIHS CoS development. Subsequent updates and future enhancements are out of the scope of this work.

The E-RIHS CoS provides a single-entry point to explore physical and digital services organized into four platforms: ARCHLAB (scientific archives), FIXLAB (fixed laboratories), MOLAB (mobile laboratories), and DIGILAB (digital data and tools). The CoS supports E-RIHS’s mission to enable Open Science practices and foster interoperability among research infrastructures around Europe and beyond. Additionally, by integrating a semantic metadata model, natural language search capabilities, and a system of dashboards, it offers a streamlined and FAIR-compliant pathway from service discovery to access implementation.

## Background for the development of E-RIHS CoS

Several RIs in Europe have developed digital CoS, and marketplaces to provide researchers with access to scientific services, datasets, and software. These platforms enhance resource visibility and usability while exploiting the benefits of data reusability and advanced analytical technologies. To this aim, one of the most challenging aspects of creating a reliable CoS, suitable for a wide range of potential users, is setting interoperable systems, and standardised and normalised metadata concerning datasets and services. ^11^

To assess the current landscape within RIs and to address the challenges associated with developing a heritage science CoS, a mapping of existing solutions at the European level was undertaken starting from the E-RIHS PP project (2017–2020, G.A. 739503). ^12^ The mapping employed two complementary methodologies: direct interviews with RIs managers and hands-on testing of other catalogues from the user perspective. Although structurally different from traditional CoS, marketplaces were also included to provide comparative insights. In the ESFRI landmark, E-RIHS is one of the 11 RIs in the Social Science and Humanities domain. Among these, only two offer physical services in addition to digital resources. Besides, E-RIHS is the only one that currently primarily offers access to physical services.

Among the different catalogues revised, several well-developed CoS that support access to both physical and digital services were identified for an in-depth examination: ELIXIR, ^13^ and CORBEL, ^14^ in the life sciences domain, the Virtual Unified Office of CERIC-ERIC, ^15^ in materials science, CESSDA in social sciences, ^16^ and the ELVIS portal of DISSCo in biodiversity research. ^17^ These CoS include advanced functionalities such as proposal management, reviewer assignment, and service usage tracking, capabilities that are essential also for E-RIHS.

Another example of advanced CoS represents the ARIA software developed by Instruct-ERIC, a European RI in the biology field, a cloud-based tool that facilitates access to the RIs’ scientific services. It is currently used by at least nine RIs, demonstrating its versatility. ^18^ However, the unique complexity and interdisciplinary nature of the heritage science domain required the development of a tailored model to effectively support the specific requirements of scientific services within E-RIHS.

Furthermore, the interviews with the CoS managers/developers provided key insights into the underlying workflows, as well as the processes used to manage evaluation of the research proposals. For example, the dashboard system employed by ARIA software allows for managing different proposal evaluations and monitoring the project visit in the premises of the host institution, while facilitating the communication among all the actors involved.

The following CoS key characteristics were identified for its successful implementation: a) adequate service description, b) metadata quality, c) harmonised data structure, d) user interface clarity, and e) search functionalities to facilitate discoverability. In this respect, the first E-RIHS CoS was developed during the E-RIHS Preparatory Phase (2017–2020), marking a significant step forward for the heritage science community in creating a dynamic, online catalogue.

The initial conceptual data model was informed by the knowledge and outcomes of previous European projects – particularly PARTHENOS, ^19^ IPERION CH, ^20^ and ARIADNE. ^21, 22^ It was implemented as a Relational Database Management System, developed in SQL technology, and represented a major improvement over existing tools and frameworks available at the time for managing heritage science services. ^12^ One of the main challenges was to structure the database to reflect the multi-valued attributes to conform the specific entities created to cover the extensive information at the base of the CoS. Over the last few years, a dedicated coordination group within the heritage science community has defined a shared conceptual framework for service description, metadata representation, and data structuring. This framework was iteratively discussed and refined in close cooperation with service managers (then referred to as “providers”) responsible for the delivery of individual services, ensuring both domain relevance and operational feasibility.

The first online catalogue, conceived as a sort of e-commerce website, was released during the IPERION HS project in 2020. ^23^ In coordination with the ARCHLAB, FIXLAB, and MOLAB platform leaders, services were clustered into high-level functional categories (e.g. spectroscopic analysis, spot analysis, etc.), leading to the formalisation of a coherent data model. ^12^ In the absence of a consolidated domain ontology or standardized controlled vocabularies for heritage science, service managers were invited to populate the model fields and contribute domain-specific terminology reflecting established practices within their scientific communities. The coordination group subsequently carried out an extensive, tricky process of data curation, harmonisation, and standardisation, validating semantic consistency and structural coherence across the catalogue. The scientific services offered by 100 institutions were delivered through three platforms: ARCHLAB, FIXLAB, and MOLAB. Users and service managers tested the first version of the catalogue, allowing for fixing bugs and unclear submission processes, and criticalities for future improvements were identified and registered.

**Figure 1.**
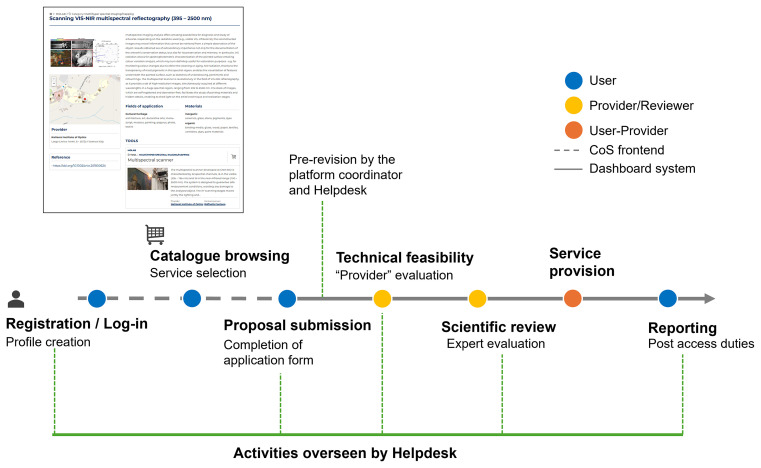
Scheme of the CoS system released during the IPERION HS project. The different phases and actors involved are indicated.

The process to access the scientific services is illustrated in Figure 1: users create a profile, then browse the catalogue to select the most adequate service(s) that best match their research needs, and finally submit a proposal. Successful proposals were granted based on the technical feasibility (evaluated by the provider) and subsequently on scientific excellence, reviewed by an international and independent panel of experts. A system of dashboards in the CoS backend was created with different levels of permission according to specific roles: platform coordinator, helpdesk, provider, and user. All notifications were managed through an automatic email system throughout the entire process. Finally, the user was responsible for completing two surveys and uploading a report (referred to as “post-access duties”). All data and files could be downloaded and analysed to monitor the IPERION HS access performance.

Despite its pioneering role, the platform developed within E-RIHS PP and IPERION HS faced challenges related to lack of metadata standardization, user interface complexity, and scalability. For example, many scientific techniques included in the catalogue had no standard designation, and scientists used different ways to refer to the same instrumentation. Similar issues were connected with complex methodologies or the targeted materials investigated by a single technique. All these factors made the selection of the most adequate service for a non-expert difficult.

Building on the experience of IPERION HS and informed by the launch of the SSHOC and EOSC marketplaces in 2021 and 2022, respectively, the E-RIHS community undertook an update of its CoS model starting from 2022. This revision aligned the model with emerging interoperability and FAIR data standards, while integrating innovative features aimed at improving service discoverability and enhancing the user experience. The redesign of the data model was made possible through regular meetings of a group dedicated to the catalogue’s development, during which different perspectives were discussed and iterative revisions were necessary. The users’ point of view was also considered and was included via targeted questionnaires distributed to experienced users and, more broadly, to the heritage science community through dedicated mailing lists. The resulting new E-RIHS CoS draws upon the heritage science community’s prior work and incorporates key advancements from other research infrastructures, delivering a more robust, interoperable, and user-friendly system.

## FAIR-by-design methodology

ESFRI has emphasized the central role of RIs in advancing Open Science, specifically advocating for the adoption of FAIR principles and the provision of quality-assured open data. According to ESFRI, ^24^ RIs are key actors in driving cultural change, enhancing data quality, and broadening access within the evolving EOSC ecosystem.

In this framework, E-RIHS has developed its CoS in alignment with this vision, FAIR-by-design and fully interoperable with other RIs marketplaces in other scientific fields, such as EOSC, SSHOC, and H2IOSC, acting both as a data and service provider. It enables seamless data integration and discovery, fostering the practice of Open Science in heritage science. This is achieved through the curation of service data displayed, facilitating the findability and interoperability. Each service is described using a detailed metadata schema that includes technical specifications, associated platforms, hosting institutions, service managers, and relevant references. The embedded policies and tools, such as machine-actionable Data Management Plans (DMPs), Creative Commons licensing, and the assignment of persistent identifier systems contribute to ensure metadata quality. The FAIR compliance of the features of the new E-RIHS CoS is summarized in the Table below ( Table 1).

**Table 1.** Key components of the E-RIHS CoS and the relative FAIR principles. F = Findable, A = Accessible, I = Interoperable, R = Reusable. Details on the FAIR principles are reported in GO FAIR. ^25^^–^^27^

CoS component	FAIR principle
Structured and semantically enriched metadata	F2, F3, I1, R1
Data indexed via a semantic engine powered by Elasticsearch	F1, R1
Free text search powered by Elasticsearch	F3
Unique persistent identifiers for data	F1, F3
Existence of Open APIs	A1
Controlled vocabularies and multilingual thesauri (developed in OpenTheso)	I2, R1.3
Cross-platform and cross-community service discovery	F4
Collaborative dashboard with a different access rights system	A1
Secure single sign-on system (e.g. ORCID)	F1
JSON-based schemas that model entities (service, person, organisation, technique, tool, method, KPIs)	I1, I3
Adherence to EOSC and CORDRA standards	I1
Data ingestion and exchange with external platforms (EOSC, SSHOC, H2IOSC)	F4
Machine-actionable metadata, knowledge representation, and harmonisation	F2
Research outputs (Reports, Datasets) available on Zenodo	F1
Research outputs identification via DOI	F1, F3, I3
Open-source software	R1.1
Data export in different formats	I1

All these elements reflect the new approach to a “research need-oriented catalogue”. ^28^ Through rigorous schema design, semantic enrichment, federated authentication, and open dissemination mechanisms, E-RIHS facilitates Open Science practices and promotes reproducibility, transparency, and collaboration in heritage science research.

## Architecture, structure, and functions of the system

The development of the E-RIHS CoS was preceded by the creation of its digital environment. A dedicated GitHub workspace, ^28– 30^ hosts schemas ( Table 2) and documentation, while a metadata knowledge base, ^31^ powered by CORDRA. ^32^ serves as the central repository for service-related digital objects.

**Table 2.** List of the schemas available on GitHub. ^28^

Schema	Description
Service	This schema is intended to model the metadata and details required to document and describe service or access providers within E-RIHS. These services will be offered by one or more funding programmes and exploited in one or more research projects.
Organisation	It represents the research institution, museum, etc., to which one or more persons can be affiliated. It contains information regarding the name, address, geographical coordinates, website, etc.
Person	It represents a person affiliated with a specific organisation. It contains information such as name, biography, country, role, etc. The role is particularly important since it defines who will be contacted (Contact person or Service Manager) when a User selects a service.
Technique	This schema is intended to model the metadata and details required to document and describe techniques that are used within access offerings in E-RIHS.
Tool/Equipment/Software	It represents the Equipment, Tool or Software from more than one equipment employed. It describes the potential results obtained (output).
Method	It describes the procedure on how the Tool/Equipment/Software is set-up and used. It is expected to be a default standardized description reusable to define the service offered by different Service Managers.
KPI	It represents the Equipment, Tool or tool form from more than one equipment employed. It describes the potential results obtained (output).

**Figure 2.**
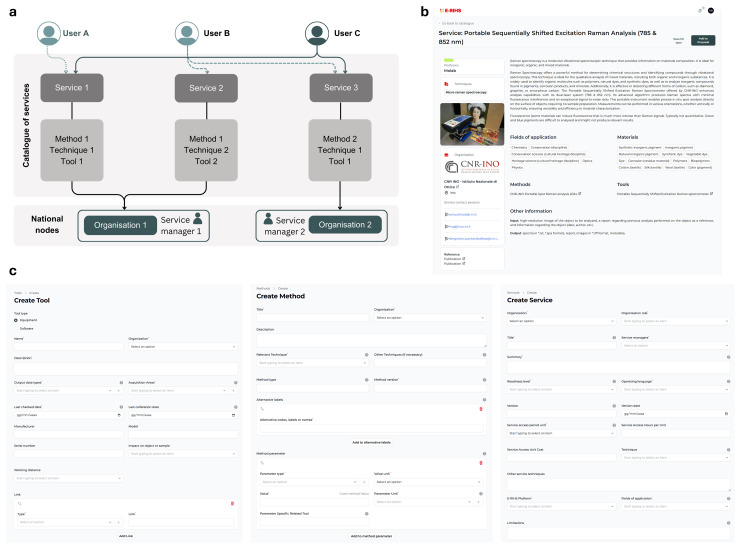
a) Simplified schema of the architecture of the E-RIHS CoS and its interaction with the potential users. The frontend of a service included in the CoS is shown in b) along with the dashboard system for compiling its metadata in c).

The data model for the CoS is based on the knowledge based created for E-RIHS. ^33^ A selection of metadata fields was considered in order to respond to the requirements of the model centered on the service entity ( Figure 2a–b), which acts as the main aggregation point and entry within the CoS. Each service is linked to one provider (i.e. an organization and a service managers) through explicit object properties that define responsibility for its implementation and delivery. Services are also associated with one or more platforms, representing the operational environments where they run, as well as with one or more tools and methods entities that describe the scientific approaches and technical capabilities they offer. These relationships ensure semantic consistency, interoperability, and machine-actionable service descriptions.

The inclusion of a service in the CoS aligns with the broader E-RIHS access policy and infrastructure governance, ^34^ which defines the eligibility criteria for platforms and services within the RI’s operational framework. Services enter the E-RIHS CoS through a provider-driven submission process and must demonstrate compliance with the relevant access and platform eligibility requirements. Service information is then populated via the dashboard system ( Figure 2c), which captures all required metadata fields, followed by editorial and metadata-quality validation by the User Helpdesk. An ongoing review and integration process ensures consistency and homogeneity across service records. After initial inclusion, services remain subject to regular review, managed through an internal versioning system. This workflow ensures that new or evolving service offerings, for example as newly developed techniques or digital tools can be added and updated over time in response to technological advancements and the needs of service managers.

An innovative addition with respect to the first CoS model implemented during IPERION HS is represented by the “Method” schema. This new entity captures the procedural knowledge required to conduct services across different service managers. The “Method” describes the way a specific equipment is employed by a service manager to offer a particular service. This could be the same for different service managers ( Figure 2), yet its preparation needs a certain level of standardisation and the designation of the actor or institution in charge of the definition of this entity. This schema enhances reproducibility and supports the harmonisation of service offerings across the E-RIHS network.

The combination of the schemas allows for a modular and scalable representation of services. The OpenTheso-based vocabulary platform – a multilingual and multi-hierarchical thesaurus manager that follows the ISO-25964 standard, ^35, 36^ – further supports multilingualism and semantic clarity. Its integration with the CoS via open APIs ensures dynamic vocabulary management.

The new architecture and the use of open APIs increased interoperability with other tools developed within E-RIHS (DIGILAB) and with systems created in the context of other research infrastructures or projects at European and national level (e.g. EOSC, SSHOC, Resinfra, H2IOSC). As a result, all the data in the E-RIHS catalogue can be easily harvested and reused by the European marketplaces, expanding its accessibility. Furthermore, the catalogue system is fully released as open-source software, enabling its reuse and adaptation by other research infrastructures.

This CoS has been developed following two main guiding principles: responsiveness to the research needs of users and a co-creation approach involving service managers, users, and ICT experts. This marks a strategic transition from a “technique-oriented” to a “research need-oriented” catalogue. A key aspect was the creation of an independent platform built on a scalable, modular architecture, in collaboration with Net7 ICT company, ^37^ which is particularly important for a domain in constant change and for an RI in its initial operational phase. This design allows the platform to adapt and grow with the evolving needs of the RI and can be integrated with new features and emerging technologies. The E-RIHS CoS *scalability* resides in its core architectural and conceptual requirements since it is designed to accommodate growth in users, service records, and access requests without performance degradation, while supporting the progressive extension of functionalities through modular components and open APIs. At the same time, the data and semantic levels are handled through an extensible data model and controlled vocabularies that can evolve without compromising coherence or interoperability, facilitating the inclusion of new services originating from disciplines progressively incorporated into the scientific community, such as architecture, bioarchaeology, and related domains.

The architecture and requirements were defined through extensive consultations with stakeholders, including researchers, service managers, and infrastructure administrators. Interviews and iterative user experience (UX) testing cycles enabled the project team to identify key pain points and user expectations. The insights gained informed the creation of a detailed specification document that guided system development.

The new E-RIHS CoS. ^38, 39^ is a standalone platform composed of two primary components: (1) a frontend interface serving as the entry point for users, and (2) a backend system dedicated to managing and monitoring proposals and access activities. Its implementation relied on contemporary web technologies, including the Laravel PHP framework for the backend and the Elasticsearch engine for semantic and faceted search. Laravel supports a robust, maintainable codebase including role-based access, workflow automation, and secure data handling. On the other hand, Elasticsearch enables robust and scalable search functionality, combining natural language processing with structured filtering. It also supports high-performance querying across large datasets, delivering fast and precise results essential for navigating extensive research service catalogues.

Advanced filters have been incorporated into the search functionality, to filter services for example, by type, research domain, geographic location, and availability. This feature enables users to precisely target relevant services, significantly improving search efficiency and user experience. The Elasticsearch-based implementation fosters an intuitive discovery process, even for unfamiliar users employing specific keywords or terminology and ensures that the system can scale effectively as the number of services and users grows, maintaining consistent performance and reliability.

**Figure 3.**
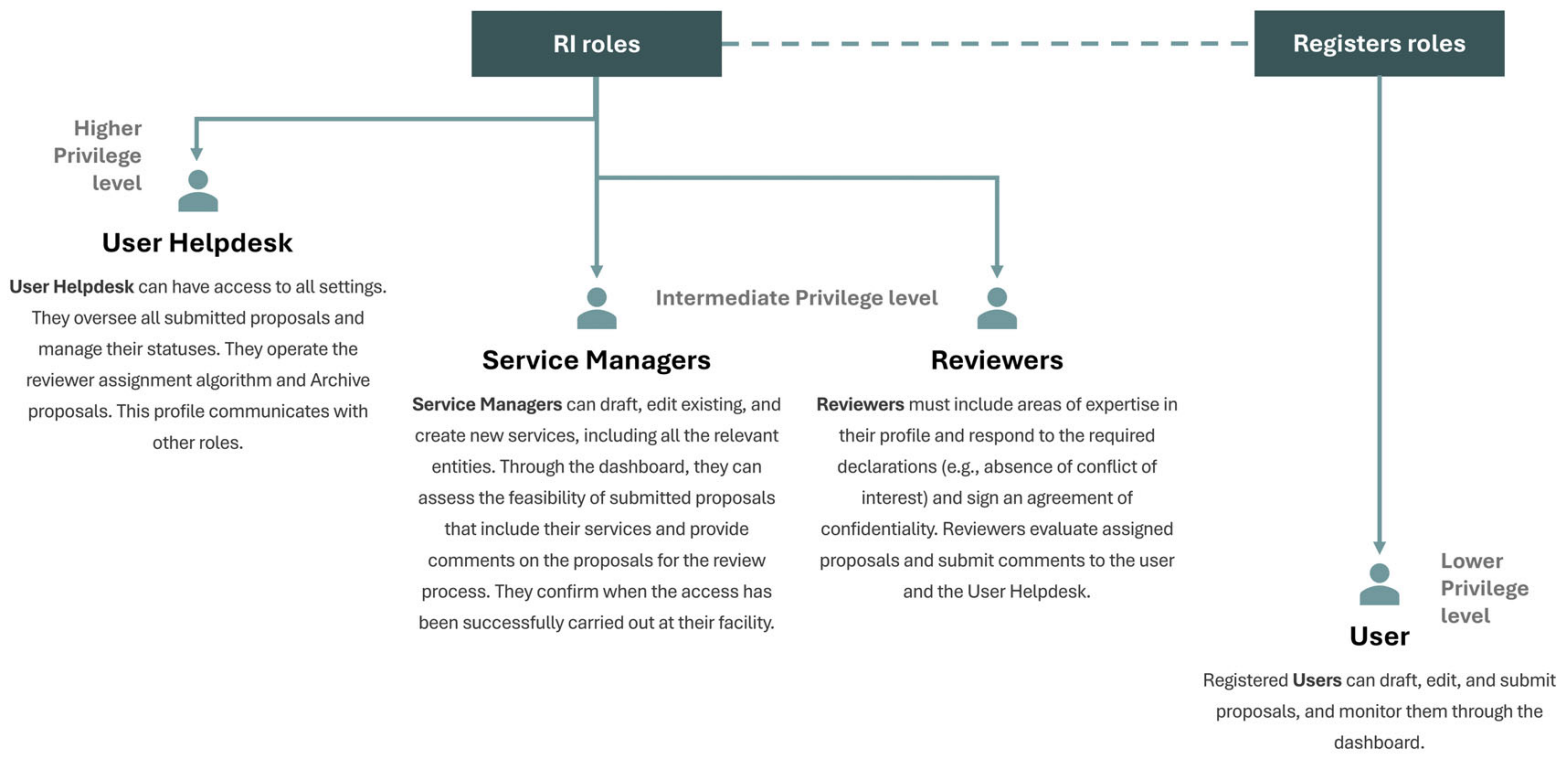
List of roles involved in the E-RIHS CoS and their corresponding dashboard functionalities.

The CoS involves four key actors ( Figure 3): the User, the Service Manager (previously “provider”), the Reviewer, and the User Helpdesk. Access level varies according to the role and the dashboard system is tailored for each actor. The User Helpdesk is responsible for overseeing and managing the entire process, from submission to post-access activities, and for assigning roles to the other actors involved.

**Figure 4.**
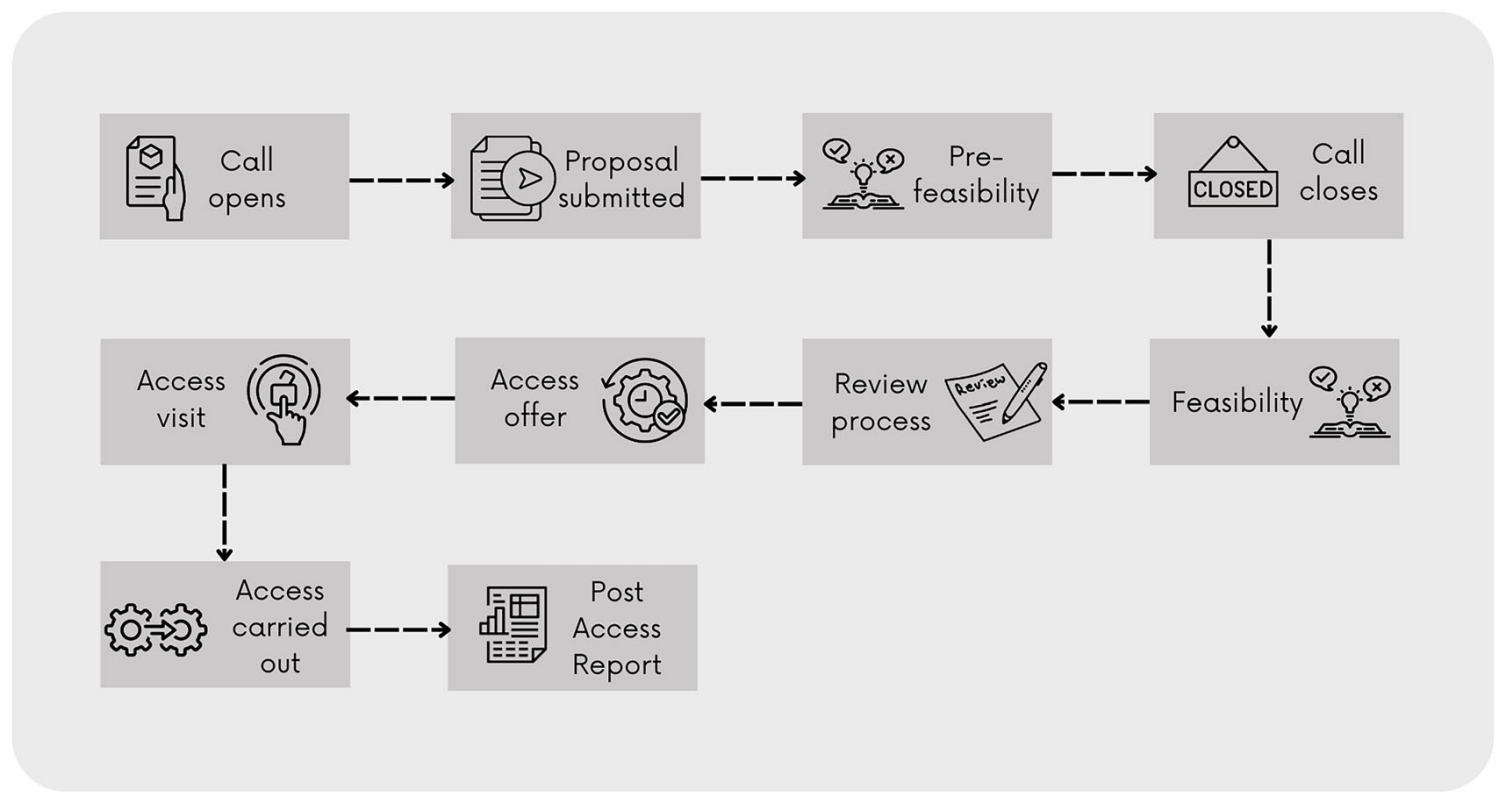
CoS workflow as defined by the E-RIHS Access Policy. ^40^

Behind the CoS, a detailed workflow ( Figure 4) was embedded in the system and reflects the access model. ^40^ defined by E-RIHS: proposals are submitted through periodic cut-off calls, undergo technical feasibility checks by service managers, and are subsequently evaluated by independent reviewers.

The procedure is simple; users can browse the catalogue, even without creating a profile, which makes the service information open. To select services and add them to a research proposal, the users must register and log in, enrich their profile with detailed information on their scientific background and experience, fill down the online application form, and submit it within the deadline of the call for proposals.

The on-line form guides users through the proposal process and functions as a metadata generator. It feeds both the backend analytics, by extracting performance metrics, and the recommendation algorithm, by capturing comprehensive data on research needs, access requirements, and project contexts. Once the application has been submitted, service managers assess the proposal's feasibility. This step represents a co-creation phase between users and service managers, since the service manager can provide comments to improve the proposal. Should the application be partially feasible or unfeasible, it returns to the draft phase, and users are encouraged to improve the proposal, change, or remove services, and resubmit the application before the established deadline. Feasible proposals undergo a review phase. An algorithm within the CoS system selects three reviewers based on the declared areas of expertise in the metadata of the profiles and the keywords of the proposal. Reviewers score the application based on defined criteria and provide comments to the users. Top-rated applications are granted access to the services. Following this phase, users and service managers agree on the access modalities and timeline, carry out the access, and collaboratively discuss and interpret data and results. At the end of the process, users are required to submit a final report.

The dashboard system helps monitor the entire process from the submission of a proposal to the post-access duties and gathers useful information that can be used to improve the CoS system and user experience. Their design focused on usability and intuitiveness, offering users a streamlined experience that makes it easy to access all key features. To this aim, a system of tabs is used to browse different pages where all the components are stored, such as the list of applications, the list of documents, and the application history.

As outlined in the E-RIHS Access Policy, ^34^ the RI provides continuous access through open calls for excellence-driven access, by ranking the proposals submitted and guaranteeing the maintenance of high-level standards. Two cut-off deadlines are expected by the year, one every six months. Additionally, the system can manage also market-driven and fast-track accesses.

At the time this work was developed (2020-2025), a major innovation in development was the integration of a recommendation engine within the CoS, inspired by recommender systems used in e-commerce and media platforms. This AI driven component was designed to support users in identifying the most appropriate services based on their research needs, having in mind that the community is broad and that not all users are experts in the scientific fields or equipment characteristics and methodological applications. The system is based on a Retrieval-Augmented Generation (RAG) architecture that integrates language models with a vector indexing infrastructure. ^41^ By generating high-dimensional embeddings and leveraging a vector database (Elasticsearch), the system performs similarity search operations to semantically map user queries to indexed content. The various strategies adopted – ranging from classical semantic similarity to multi-vector and multi-querying approaches – enable increased contextual granularity, improved robustness of information retrieval, and mitigation of model hallucination phenomena.

The engine was designed to analyse a broad array of data, including equipment characteristics, methodological applications, past project outcomes, and user feedback, to generate personalized recommendations. Natural language queries are supported to increase accessibility and inclusiveness, especially for non-experienced users looking for the best solution for their research question. However, the AI-driven recommendation engine is not intended to replace the expertise of heritage scientists or the fundamental relationship between users and service managers. Rather, it serves as an initial orientation tool to guide users through the complex landscape of services and to build a more structured data foundation for this multifaceted sector. Data for training the algorithm are being collected through pilot applications distributed among the community and from E-RIHS service managers, with all processing conducted in full compliance with EU data protection regulations. Additionally, a dedicated working group identified representative research needs, compiling standardized controlled vocabularies and classification schemes to be used in service descriptions.

The contribution of researchers has been critical in enhancing the robustness and reliability of the system. Questions automatically generated by the Large Language Model (LLM) underwent manual validation, transforming a synthetic dataset into a golden dataset of reference, essential for rigorous endpoint evaluation. This intervention significantly impacted not only the improvement of retrieval quality but also the establishment of a structured feedback framework, which is fundamental for calibrating embedding techniques and optimizing the entire RAG pipeline according to E-RIHS’s operational requirements.

The effectiveness of the recommendation engine depends on the quality and volume of structured metadata collected via the application form and backend monitoring tools. Users’ engagement through input and feedback directly contributes to refining the system, allowing it to function as a dynamic, self-improving resource that supports decision-making, increases efficiency, and enhances the scientific impact of access activities. In a context marked by the rapid evolution of AI-driven technologies, targeted financial investments will be necessary to ensure future system updates and scalability.

## Current state of the CoS

**Figure 5.**
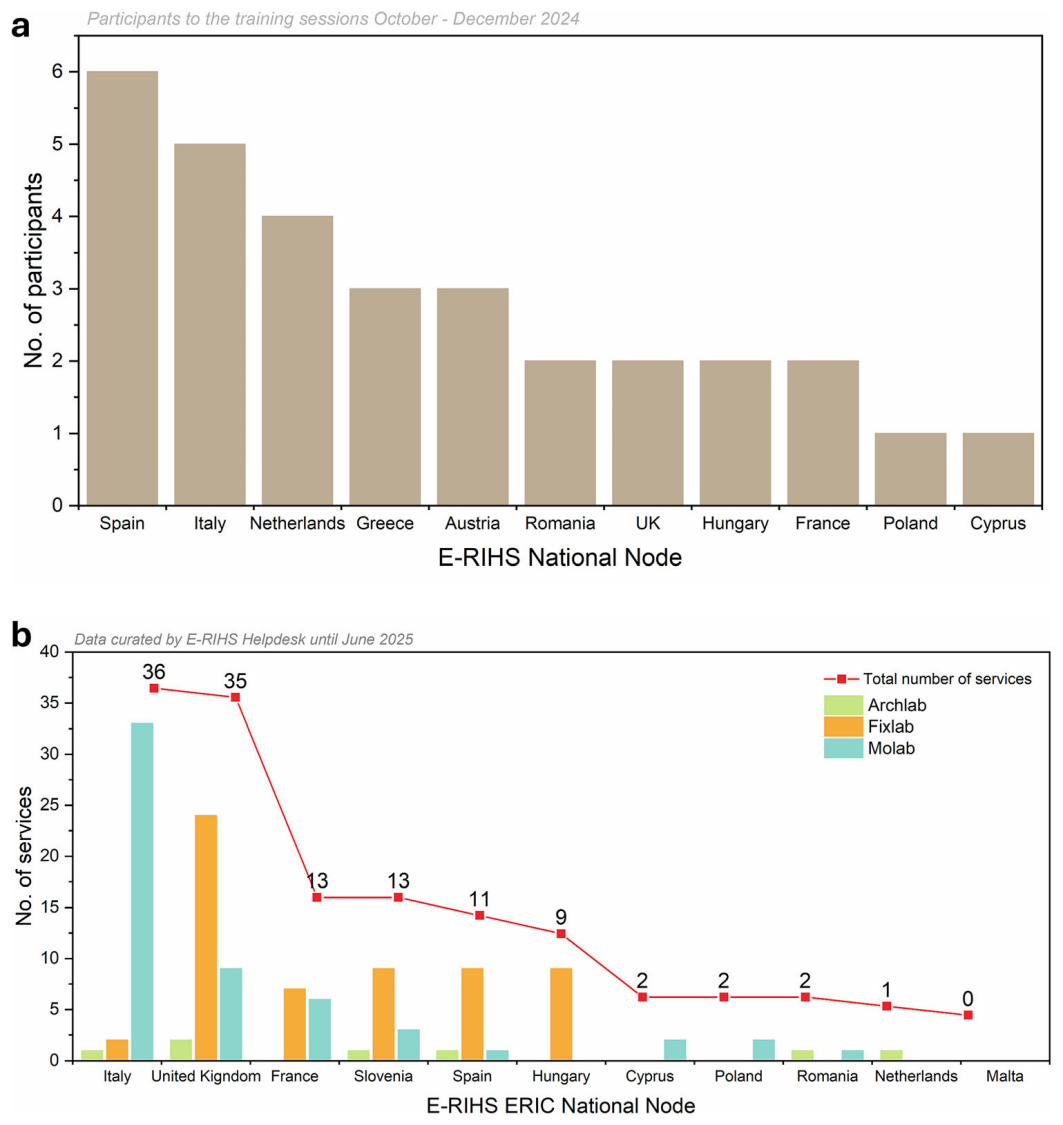
a) Participants from E-RIHS nodes trained; b) E-RIHS ERIC Curated services catalogue across three platforms (by June 2025).

The new E-RIHS CoS platform marked a milestone in delivering integrated access to heritage science services. Its first version, including the dashboard system, was released in October 2024. This release followed an intensive phase of design, prototyping, testing, and iteration, culminating in the transition from development to operational deployment. From the earliest stages, service managers were engaged in the co-design of the system, contributing to data modelling, the selection of controlled vocabularies, and the definition of access procedures. To support the operational rollout, training sessions for service managers of the different platforms were conducted from October to December 2024, involving a total of 32 representatives from E-RIHS National Nodes to start inserting services ( Figure 5). They also served as validation environments, highlighting formal and functional requirements. Among the issues and changes identified in this phase can be listed the inclusion of a comment section from service managers to users following the feasibility evaluation to enhance the co-creation and collaboration between the user and the scientists. Also, the specialist involved identified the need to enable proposal revision and resubmission which required a revision in the functioning workflow.

The sessions, which were recorded and distributed to reach a wider audience, also allowed to identify concepts that were not completely clear to the service managers and represented an opportunity to adjust, when necessary, and explain the data model at the foundation of the CoS. Moreover, guidelines were prepared and made publicly accessible through the E-RIHS Zenodo community. ^40, 42, 43^ In parallel, technical support facilitated user onboarding and encouraged early engagement with the platform. At the moment this work was written (June 2025), 124 services are included in the CoS across three of the four platforms, involving ten E-RIHS national nodes ( Figure 5). Their data are under revision by the Users Helpdesk to ensure consistency and homogeneity, a process that will be constantly and regularly updated. Also, digital services from the DIGILAB platform are progressively integrated through APIs to allow their discoverability.

Intermediate testing phases involved a significant number of participants, covering multiple dimensions of the user experience, such as intuitiveness of the dashboard, filtering and search mechanisms in the catalogue, as well as the effectiveness of the proposal submission workflow. Reviewers and the User Helpdesk assessed the peer-review system, focusing on proposal evaluation and approval processes. Several users (>50) were interviewed on usability, performance, and overall satisfaction, generating both qualitative and quantitative insights that directly informed development cycles.

The iterative UX testing approach resulted in concrete enhancements across the platform. Navigation structures were improved, visual elements were optimized, and user journeys were made more intuitive. To ensure methodological coherence in user-centred design, the IT company formally integrated a professional UX designer into the development team, supporting evidence-based design decisions and consistency across interfaces. UX testing was conducted in multiple iterative sessions involving different user profiles: (i) expert users already familiar with the IPERION HS catalogue, engaged to optimise task efficiency and reduce interaction latency; (ii) heritage science practitioners without prior exposure to the catalogue, involved to assess general usability and cognitive load; and (iii) non-specialist users with no previous experience of the system, recruited to evaluate navigability, onboarding effectiveness, and critical friction points within the access workflow. System performance and security were validated through comprehensive testing, including penetration tests and code reviews. Feedback collected through UX testing and real-time usage analytics continues to inform system updates. The co-creation philosophy embedded in the development process has proven essential for achieving a usable and adaptable platform.

To support continuous monitoring and decision-making, the CoS includes a Business Intelligence tool integrated into the User Helpdesk dashboard. This tool enables KPIs tracking and supports strategy adjustments based on platform usage data. The analytics module provides detailed insights across several dimensions:

• Proposals: total number and number per call/country; acceptance and reserve list rates; gender-disaggregated data; type classification (new, long-term, resubmission); status tracking.
• Scientific Disciplines: submission and acceptance breakdowns by discipline.
• Users: total number of users; submission activity; user-to-proposal ratios.
• Tools: request frequency per tool and call.

The resulting data can be visualised through graphs and maps and is available for download. Regular updates, based on the iterative engagement process with users and service managers, ensure that CoS remains aligned with the expectations and workflows of its users, consolidating its role as a central component in E-RIHS access provision.

## Conclusions and future perspectives

The development of the E-RIHS CoS demonstrates how a service-oriented digital platform can be used to structure and formalise the knowledge of a highly interdisciplinary research community through shared standards, common conceptual frameworks, controlled vocabularies, and FAIR-aligned data practices. Beyond the management of access to the scientific services, from proposal submission to delivery, the catalogue’s workflow acts as an enabling mechanism for knowledge co-creation, fostering collaboration among researchers with diverse expertise and objectives, highlighting how an interoperable user-centered framework can overcome disciplinary barriers and support sustainable and cross-domain research ecosystems for scientists and other specialists.

The E-RIHS CoS is contributing significantly to the adoption of Open Science practices within the heritage science community. Through its adherence to FAIR data principles, support for reproducible research workflows, and user-centered design, the CoS promotes accessibility, transparency, and knowledge sharing. It also fosters cross-disciplinary collaboration and co-creation, enriching research at all stages—from project planning to reporting.

The CoS stands as a flagship initiative in digital heritage science infrastructure, showcasing how modular design, AI integration, semantic interoperability, and community co-creation can converge to deliver impactful, sustainable, and inclusive research services.

The E-RIHS CoS has been designed with a modular architecture to ensure long-term extensibility and maintainability. A core area of ongoing development will concern the further refinement of an AI-based recommendation engine. Although the current version of the CoS includes basic personalised suggestions, further enhancements will leverage structured metadata and behavioural analytics to support dynamic service recommendations. Powered by machine learning and natural language processing techniques, context-aware recommendations for individual research goal will improve with previous usage patterns. This capability, powered by growing number of available training data, will enhance discoverability and increase the efficiency of service selection processes. The AI-driven metadata analysis will progressively enhance the following features:

• Automated enrichment of metadata through natural language processing.
• Resource indexing and improved catalogue navigation.
• Predictive analytics for user behaviour and resource utilisation trends.

The application of non-generative AI technologies contributes to efficient data management and consistent metadata updates, forming the backbone of a scalable and intelligent digital infrastructure. The CoS is constantly aligned with the EOSC ecosystem and other domain-specific RIs, such as CLARIN, CERIC ERIC, and DARIAH. Currently available in English; however, a multilingual expansion to support broader accessibility and inclusiveness could be implemented. To this purpose, for example in collaboration with the CLARIN RI, a multilingual metadata management and query translation could include:

• Metadata Translation: Automated and manually validated translation of metadata fields, beginning with languages of the E-RIHS National Nodes, using semantic frameworks and controlled vocabularies (e.g., SKOS, AAT, and EDM).
• Query Translation: Implementation of multilingual search capabilities using CLARIN tools to ensure terminological consistency and user-friendly query interfaces.

CLARIN’s expertise in domain-specific term extraction, translation, and controlled vocabulary creation could guide this process, ensuring high-quality, interoperable multilingual support for both human-readable and machine-actionable metadata. ^44, 45^ Moreover, the semantic annotation of resources could allow a more precise classification and linking of information across domains.

The ERIC legal framework of E-RIHS is reinforcing governance structures and long-term sustainability of the CoS. Under E-RIHS ERIC, responsibilities for catalogue maintenance, technological evolution, user support, and policy compliance has been formally allocated, ensuring continuity and accountability. This governance model supports the long-term operation and strategic development of the platform in alignment with international standards and community needs.

## Ethics and consent

Ethical approval and consent were not required.

## Abbreviations

**Table:** 

**AAT**	Art and Architecture Thesaurus
**AI**	Artificial Intelligence
**API(s)**	Application Programming Interface(s)
**ARIA**	Access to Research Infrastructure Administration
**ARIADNE**	Advanced Research Infrastructure for Archaeological Data Networking in Europe
**CERIC**	Central European Infrastructure Consortium
**CLARIN**	Common Language Resources and Technology Infrastructure
**CORBEL**	Coordinated Research Infrastructure Building Enduring Life-science services
**DARIAH**	Digital Research Infrastructure for the Arts and Humanities
**E-RIHS **	European Research Infrastructure on Heritage Science
**E-RIHS IP**	European Research Infrastructure on Heritage Science – Implementation Phase
**E-RIHS PP**	European Research Infrastructure on Heritage Science – Preparatory Phase
**EDM**	European Data Model
**ELIXIR**	European Life-science Infrastructure for Biological Information
**EOSC**	European Open Science Cloud
**ERIC**	European Research Infrastructure Consortium
**ESFRI**	European Straregy Forum on Research Infrastructures
**FAIR**	Findable, Accessible, Interoperable, Reusable
**H2IOSC**	Humanities and cultural Heritage Italian Open Science Cloud
**ICT**	Information and Communication Technology
**IPERION CH**	Integrated Platform for the European Research Infrastructure on Cultural Heritage
**IPERION HS**	Integrating Platforms for the European Research Infrastructure ON Heritage Science
**ISO**	International Organization for Standardization
**KPIs**	Key Performance Indicator(s)
**PARTHENOS**	Pooling Activities, Resources and Tools for Heritage E-research Networking, Optimization and Synergies
**ResInfra**	EU-CELAC Joint Initiative for Research and Collaboration – Research Infrastructure
**RI(s)**	Research Infrastructure(s)
**SKOS**	Simple Knowledge Organization System
**SQL**	Structured Query Language
**UX**	User Experience

## Data Availability

The E-RIHS CoS is online, available at the link: https://catalogue.e-rihs.eu/catalogue. The dataset was derived from the database of the E-RIHS ERIC Catalogue of Services and is available at Zenodo: CNR INO Dataset for the E-RIHS CoS: https://doi.org/10.5281/zenodo.16532272. ^39^ The project contains the following underlying data:
•
E-RIHS CoS_CNR INO_Dataset.xlsx The dataset is available under the terms of the Creative Commons Attribution 4.0 International license (CC-BY 4.0).

## Software availability

• Source code available from: https://github.com/net7/ERIHS_catalogue/tree/v.1.0
• Archived software available from: https://zenodo.org/records/16362229
• License: GPL v3
