# Supplementary material for: Designing a FAIR Catalogue of Services for the Heritage Science community
Source: Open Res Eur. 2026 Feb 24;5:335. Originally published 2025 Nov 3. [Version 2] doi: 10.12688/openreseurope.20798.2 (PMC12963830; doi:10.12688/openreseurope.20798.2)
Supplement: Peer review history

# Reviewer report: Reviewer response for version 2

**DOI:** 10.21956/openreseurope.24784.r70336

**Published:** 2026-06-18

**Authors:** Sonja Herres-Pawlis

**Affiliations:** 1 RWTH Aachen University, Aachen, Germany

This article describes the design, implementation, and deployment of the E-RIHS Catalogue of Services (CoS), a FAIR-compliant digital platform that serves as the main access point to scientific services offered by the European Research Infrastructure for Heritage Science (E-RIHS). The platform aims to help researchers identify, request, and manage access to heritage science facilities, archives, instruments, expertise, and digital resources.

 A central challenge addressed by the authors is that heritage science is highly interdisciplinary, bringing together chemists, physicists, conservators, archaeologists, art historians, and engineers. Many users are not experts in analytical techniques and therefore struggle to identify appropriate facilities or methods for their research questions.

 The paper traces the evolution of the catalogue from earlier E-RIHS and IPERION projects to a more sophisticated platform built around:

- FAIR and Open Science principles
- Structured metadata and controlled vocabularies
- Semantic search using Elasticsearch
- Workflow management for proposal submission and peer review
- Role-specific dashboards
- Open APIs and interoperability with EOSC and other research infrastructures
- An emerging AI-based recommendation engine to help users identify suitable services

A major conceptual shift is from a "technique-oriented" catalogue (listing instruments and analytical methods) to a "research-need-oriented" catalogue that starts from the user's scientific problem. This is really good for the community!

 The architecture is based on modular schemas (Service, Organization, Person, Technique, Tool, Method, KPI) and a knowledge-based infrastructure intended to support interoperability and future scalability. The contribution also describes the workflow through which users submit proposals, receive feasibility feedback from service managers, undergo peer review, and ultimately gain access to E-RIHS facilities.

 The strongest technical contribution is the explicit attempt to apply FAIR principles not only to datasets but also to service descriptions and service discovery. The authors provide a mapping between catalogue features and FAIR principles and describe metadata structures, identifiers, APIs, controlled vocabularies, and interoperability mechanisms.

***Small point for a potential revision:** The authors improved the table but still do not provide a systematic FAIR maturity assessment.*

 The catalogue was developed through sustained engagement with service managers, users, platform coordinators, and ICT developers over several years and I would like to point out that this iterative refinement, testing, and community involvement is crucial for a good CoS. I would like to note that heritage science includes many users who are not specialists in analytical instrumentation and that the report´s emphasis on usability, recommendation support and co-creation is therefore well justified.

 The AI recommendation engine based on RAG architecture is innovative and potentially useful for non-expert users. In my view, the authors correctly position it as a support tool rather than a replacement for expert consultation.

 This report documents a real operational system that supports a major European research infrastructure, and the practical impact is evident. The catalogue reflects nearly a decade of development work, beginning with E-RIHS Preparatory Phase and IPERION HS. Hereby, the report captures valuable lessons from infrastructure-building that are rarely documented systematically.

 The platform's code, metadata schemas, datasets, and documentation are openly available. This increases reproducibility and allows reuse by other infrastructures.

 In summary, this is a valuable and timely infrastructure report documenting the creation of an important FAIR-oriented service platform for heritage science. Its major strength lies in its practical implementation and community-driven design.

If any, are all the source data and materials underlying the results available?

Yes

Is the work original in terms of material and argument?

Yes

Is the work clearly and cogently presented?

Yes

Is the argument persuasive and supported by evidence?

Yes

Does the research article contribute to the cultural, historical, social understanding of the field?

Yes

Does it sufficiently engage with relevant methodologies and secondary literature on the topic?

Yes

Reviewer Expertise:

Chemistry, FAIR data, Research data management

I confirm that I have read this submission and believe that I have an appropriate level of expertise to confirm that it is of an acceptable scientific standard.

# Reviewer report: Reviewer response for version 2

**DOI:** 10.21956/openreseurope.24784.r70270

**Published:** 2026-03-05

**Authors:** Katrina Exter

**Affiliations:** 1 Flanders Marine Institute (VLIZ), Oostende, Belgium

The authors have improved the manuscript and I have no further comments to make on the manuscript: fully approved.

 As an FYI to the authors, I would again suggest that they exposed their schemas on GH as json-ld as well (which they did say they were considering), as this will make those schemas interoperable wtih the wider research (or any other) community...following, for example, the ODIS (Ocean Data Information System) approach of schema.org+json-ld, consideing mapping to CodeMeta for software schemas, etc.   

 Good luck with future evolutions.

If any, are all the source data and materials underlying the results available?

Yes

Is the work original in terms of material and argument?

Yes

Is the work clearly and cogently presented?

Yes

Is the argument persuasive and supported by evidence?

Yes

Does the research article contribute to the cultural, historical, social understanding of the field?

Partly

Does it sufficiently engage with relevant methodologies and secondary literature on the topic?

Yes

Reviewer Expertise:

Open science, data management

I confirm that I have read this submission and believe that I have an appropriate level of expertise to confirm that it is of an acceptable scientific standard.

# Reviewer report: Reviewer response for version 1

**DOI:** 10.21956/openreseurope.22502.r63546

**Published:** 2026-01-06

**Authors:** Brian Matthews

**Affiliations:** 1 STFC, Rutherford Appleton Laboratory, Didcot, Oxfordshire, UK

The article is well-written and a good summary of the work undertaken to implement the E-RIHS CoS.

 However, I find the title of the paper misleading as the emphasis of the paper is not the design  of the Catalogue itself, but rather how it supports users in a proposal submission, evaluation  and service access workflow.  This is broader than the Catalogue itself and would thus be of interest to a different audience - service managers rather than catalogue developers. This is nicely presented and of value in itself, but as a consequence how services are actually catalogued, how service metadata is chosen and presented in the system, and how in particular new services are onboarded are only briefly touched upon. A fuller treatment of this would give better insight into the scope and usability of the system. 

 Table 1 is curious and I am not convinced that all features are correctly classified against the FAIR criteria. Particularly some of the Accessibility criteria seem misplaced.  Perhaps a more detailed assessment against the specific criteria would be of value.  Have the authors undertaken a FAIR evaluation? 

 Figure 3 seems to take a lot of space - could this be made more compact?  I am not certain of the meaning 

 of the Access Level arrow from - to +.

 The use of AI for recommendation services is interesting, but is brief and seems an afterthought. 

 Is it in response to user demand?  Do users find it hard to navigate around the system - currently 

 124 services are included - significant but not overwhelming.  Could search be enhanced in other ways?  (better keywords, user reviews and recommendations etc).

If any, are all the source data and materials underlying the results available?

Yes

Is the work original in terms of material and argument?

Partly

Is the work clearly and cogently presented?

Yes

Is the argument persuasive and supported by evidence?

Yes

Does the research article contribute to the cultural, historical, social understanding of the field?

Partly

Does it sufficiently engage with relevant methodologies and secondary literature on the topic?

Yes

Reviewer Expertise:

Data management, metadata, data infrasructure, data catalogues, FAIR research, workflows, service management

I confirm that I have read this submission and believe that I have an appropriate level of expertise to confirm that it is of an acceptable scientific standard, however I have significant reservations, as outlined above.

# Response

**Published:** 2026-01-21

**Authors:** Laura Benassi

We thank you for the careful reading of the manuscript and for the constructive comments, which helped us to better clarify the scope, focus, and presentation of the work.

**Request 1**: *This is nicely presented and of value in itself, but as a consequence how services are actually catalogued, how service metadata is chosen and presented in the system, and how in particular new services are onboarded are only briefly touched upon. A fuller treatment of this would give better insight into the scope and usability of the system.*

**Answer**: We acknowledge your observation that the manuscript places strong emphasis on how the Catalogue supports proposal submission, evaluation, and service access workflows, rather than focusing exclusively on catalogue design in a narrow sense. This emphasis was intentional. That said, we agree that aspects such as service cataloguing, metadata selection, and onboarding of new services. We have added additional information to provide better insight into how services are described, harmonised, and operationally integrated into the system, while maintaining the workflow-oriented perspective of the paper. Also, we have modified Figure 2 to include a visual representation of the dashboard system used to populate the service metadata. p.3 “Interdisciplinarity is a persistent challenge in the heritage science domain due to the different methods and languages employed to analyse, study, interpret, and intervene in cultural and natural heritage. The E-RIHS scientific community has worked to overcome the fragmentation of different approaches by creating three integrated platforms to offer its services: •           ARCHLAB, providing access to scientific archives and collections of heritage-related data, including access to samples, datasets, or scientific reports from historical or artistic artefacts preserved in scientific archives [2]; •           FIXLAB, offering large and medium-scale cutting-edge laboratory instrumentation, such as synchrotrons or particle accelerators, to analyse samples coming from the artefacts under investigation [3]; •           MOLAB, enabling the use of mobile instrumentation for on-site heritage non-invasive analysis, such as the study of an artwork directly inside the museum exhibition room [4]. Many online catalogues of services and marketplaces created by European and non-European research infrastructures primarily focus on digital tools, data, and related services. The E-RIHS CoS represents one of the few initiatives that effectively aligns services with user needs for access to archives, laboratories, and instrumentation. By prioritizing physical access, this approach introduces additional conceptual and operational complexity in both the design and implementation of the catalogue. To complement this framework with digital resources, E-RIHS is developing DIGILAB, a dedicated platform for digital tools and data that will provide a comprehensive, FAIR-compliant knowledge base for heritage digital twins.” p.5: “In coordination with the ARCHLAB, FIXLAB, and MOLAB platform leaders, services were clustered into high-level functional categories (e.g. spectroscopic analysis, spot analysis, etc.), leading to the formalisation of a coherent data model [5]. In the absence of a consolidated domain ontology or standardized controlled vocabularies for heritage science, service managers were invited to populate the model fields and contribute domain-specific terminology reflecting established practices within their scientific communities. The coordination group subsequently carried out an extensive, tricky process of data curation, harmonisation, and standardisation, validating semantic consistency and structural coherence across the catalogue.” p.9: “The inclusion of a service in the CoS aligns with the broader E-RIHS access policy and infrastructure governance [8], which defines the eligibility criteria for platforms and services within the RI’s operational framework. Services enter the E-RIHS CoS through a provider-driven submission process and must demonstrate compliance with the relevant access and platform eligibility requirements. Service information is then populated via the dashboard system (Figure 2c), which captures all required metadata fields, followed by editorial and metadata-quality validation by the User Helpdesk. An ongoing review and integration process ensures consistency and homogeneity across service records. After initial inclusion, services remain subject to regular review, managed through an internal versioning system. This workflow ensures that new or evolving service offerings, for example as newly developed techniques or digital tools can be added and updated over time in response to technological advancements and the needs of service managers.”

**Request 2**: *Table 1 is curious and I am not convinced that all features are correctly classified against the FAIR criteria. Particularly some of the Accessibility criteria seem misplaced.  Perhaps a more detailed assessment against the specific criteria would be of value.  Have the authors undertaken a FAIR evaluation?*

**Answer**: Thank you for pointing this out. Indeed, this comment agrees with a suggestion by another reviewer. We have revised and simplified the table to make a more precise identification of the FAIR principles following the descriptions in GO FAIR: https://www.go-fair.org/fair-principles/

**Request 3**: *Figure 3 seems to take a lot of space - could this be made more compact?  I am not certain of the meaning of the Access Level arrow from - to +.*

**Answer**: Thank you for this suggestion. Figure 3 has been modified accordingly, and the contents have been summarized to improve its interpretability.

**Request 4**: *The use of AI for recommendation services is interesting, but is brief and seems an afterthought.* *Is it in response to user demand?  Do users find it hard to navigate around the system - currently 124 services are included - significant but not overwhelming.  Could search be enhanced in other ways?  (better keywords, user reviews and recommendations etc).*

**Answer**: Thank you for this comment, which again agrees with the suggestion by another reviewer.  The introduction of AI features emerged in response to the specific needs of the Heritage Science community users. We have included further information in the introduction to better contextualize the characteristics of the users involved in E-RIHS community. The challenge faced when working with a particularly heterogeneous user base, which includes not only scientists but also humanities scholars (e.g. art historians or archaeologists) who may seek scientific analyses without being familiar with available techniques or infrastructures, made it necessary to look for strategies to aid them during service selection. In this context, AI-based recommendations were intended to support user orientation and discovery, complementing search and filtering mechanisms, but not to replace human interaction. We have expanded the AI-driven system description in order to make clearer the goal of its use and characteristics.  p.14 “The new E-RIHS CoS platform marked a milestone in delivering integrated access to heritage science services. Its first version, including the dashboard system, was released in October 2024. This release followed an intensive phase of design, prototyping, testing, and iteration, culminating in the transition from development to operational deployment. From the earliest stages, service managers were engaged in the co-design of the system, contributing to data modelling, the selection of controlled vocabularies, and the definition of access procedures. To support the operational rollout, training sessions for service managers of the different platforms were conducted from October to December 2024, involving a total of 32 representatives from E-RIHS National Nodes to start inserting services (Figure 5). They also served as validation environments, highlighting formal and functional requirements. Among the issues and changes identified in this phase can be listed the inclusion of a comment section from service managers to users following the feasibility evaluation to enhance the co-creation and collaboration between the user and the scientists. Also, the specialist involved identified the need to enable proposal revision and resubmission which required a revision in the functioning workflow. The sessions, which were recorded and distributed to reach a wider audience, also allowed to identify concepts that were not completely clear to the service managers and represented an opportunity to adjust, when necessary, and explain the data model at the foundation of the CoS.”

# Reviewer report: Reviewer response for version 1

**DOI:** 10.21956/openreseurope.22502.r63538

**Published:** 2025-12-31

**Authors:** Yaxing Wei

**Affiliations:** 1 ORNL Distributed Active Archive Center, Oak Ridge, Tennessee, USA

This manuscript introduces the concept, design, and development of the new ERIHS CoS, emphasising how it follows FAIR and Open Science principles. This manuscript gives a well introduction to the co-creation process and its novelty compared with similar platforms from European RIs. It highlights the two main guiding principles that the development process followed: 1) responsiveness to the research needs of users and 2) a co-creation approach involving service managers, users, and ICT experts. The flow of the manuscript is clear and it's easy to read.

 But the manuscript can be potentially improved in multiple ways. Below are some suggestions and questions:

 As someone who is not familiar with heritage science, I found it's hard to figure out what a "service" really means in this context or what "services" are really managed in this E-RIHS CoS. Suggest to add a few sentences to introduce heritage science at the beginning, especially its broad, multi-disciplinary, and heterogeneous nature. Also, provide a high-level categorization of "services" along with some examples of each category.

 In Table 1, instead of simply using dots to indicate that a E-RIHS CoS' feature supports FAIR, be more specific and list what aspects of the FAIR principles (e.g., F1, F2, A1.1, I2, R1.3 found on https://www.go-fair.org/fair-principles/) are supported by each main CoS feature. This will better elaborate how the E-RIHS CoS supports FAIR and also help to identify gaps, if there is any.

 How are the different schemas listed in Table 2 connected with each other to form an integrated data model?

 How does this E-RIHS CoS fit into the overall E-RIHS system and the even broader European RIs? Are there use cases that users need to leverage E-RIHS CoS and other components of E-RIHS and/or European RIs to accomplish some objectives? Highlight its interoperability with other components of the E-RIHS system and/or the broader European RIs through those use cases.

 "Scalability" is listed as one of the novelties of the E-RIHS CoS. But I can't seem to find information that elaborate what "scalability" means in this context, how scalability was achieved, and if there was any effort to quantify the scalability.

 What are the lessons learned from E-RIHS CoS that can potentially benefit the broader communities, beyond heritage science and European RIs?

 The manuscript doesn't sufficiently describe how the AI-driven recommendation engine works. It says "Users’ engagement through input and feedback directly contributes to refining the system, allowing it to function as a dynamic, self-improving resource that supports decision-making, increases". But it's unclear how users' input and feedback are used to refine the system.

 The "Current state of the CoS" section described how the training sessions and intermediate testing phases were conducted and "resulted in concrete enhancements across the platform". An elaboration of what feedback was collected during the training and testing and what enhancements were made based on these feedback would be helpful.

 An Excel spreadsheet (E-RIHS CoS_CNR INO_Dataset.xlsx) is listed as a dataset associated with this manuscript. But I couldn't find this anywhere this dataset was described in the manuscript.

If any, are all the source data and materials underlying the results available?

Yes

Is the work original in terms of material and argument?

Yes

Is the work clearly and cogently presented?

Partly

Is the argument persuasive and supported by evidence?

Partly

Does the research article contribute to the cultural, historical, social understanding of the field?

Partly

Does it sufficiently engage with relevant methodologies and secondary literature on the topic?

Yes

Reviewer Expertise:

data science, remote sensing, data curation and management, geospatial information science, and environmental science

I confirm that I have read this submission and believe that I have an appropriate level of expertise to confirm that it is of an acceptable scientific standard, however I have significant reservations, as outlined above.

# Response

**Published:** 2026-01-21

**Authors:** Laura Benassi

We thank you for the positive evaluation of the manuscript, and we appreciate the detailed and constructive suggestions, which have helped us to improve the manuscript substantially. Below, we address each comment in turn.

**Request 1**: *As someone who is not familiar with heritage science, I found it's hard to figure out what a “service” really means in this context or what "services" are really managed in this E-RIHS CoS. Suggest to add a few sentences to introduce heritage science at the beginning, especially its broad, multi-disciplinary, and heterogeneous nature. Also, provide a high-level categorization of "services" along with some examples of each category.*

**Answer**: We thank you for highlighting the need to better contextualise our work. We have expanded the introductory section to describe heritage science as a broad, heterogeneous, and inherently multidisciplinary domain. pp.2-3: “Heritage Science – a broad and multidisciplinary field that focuses on the scientific study, interpretation, conservation, and preservation of cultural and natural heritage, operates across different contexts with heterogeneous domains of knowledge and stakeholders, including non-experts and the public. In the last few decades,  research on both tangible and intangible heritage has gained interest worldwide 1, 2 , drawing on a wide range of disciplines from the humanities (art history, archaeology, history), sciences (chemistry, physics, biology), and engineering (materials science, digital tools) [1]. Heritage Science has progressively strengthened its collaborative nature. These efforts to enhance scientific cooperation among the different specialists involved have led to the establishment of a dedicated European Research Infrastructure (E-RIHS).” To make the notion of “services” more concrete, we have introduced the three integrated E-RIHS platforms and provided examples of the services included for each category, including adequate examples in the reference. p.3 “Interdisciplinarity is a persistent challenge in the heritage science domain due to the different methods and languages employed to analyse, study, interpret, and intervene in cultural and natural heritage. The E-RIHS scientific community has worked to overcome the fragmentation of different approaches by creating three integrated platforms to offer its services: •           ARCHLAB, providing access to scientific archives and collections of heritage-related data, including access to samples, datasets, or scientific reports from historical or artistic artefacts preserved in scientific archives [2]; •           FIXLAB, offering large and medium-scale cutting-edge laboratory instrumentation, such as synchrotrons or particle accelerators, to analyse samples coming from the artefacts under investigation [3]; •           MOLAB, enabling the use of mobile instrumentation for on-site heritage non-invasive analysis, such as the study of an artwork directly inside the museum exhibition room [4]”.

**Request 2**: *In Table 1, instead of simply using dots to indicate that a E-RIHS CoS’ feature supports FAIR, be more specific and list what aspects of the FAIR principles (e.g., F1, F2, A1.1, I2, R1.3 found on https://www.go-fair.org/fair-principles/) are supported by each main CoS feature. This will better elaborate how the E-RIHS CoS supports FAIR and help to identify gaps, if there is any.*

**Answer**: Thank you for the valuable suggestion to make the FAIR alignment more explicit. Table 1 has been revised to indicate which specific FAIR principles (e.g. F1, F2, A1.1, I2, R1.3) are supported by each main feature of the CoS, following the FAIR principles as defined by GO FAIR.

**Request 3**: *How are the different schemas listed in Table 2 connected with each other to form an integrated data model?*

**Answer**: In response to the question on how the schemas listed in Table 2 are connected, we clarified that they are components of a unified data model, simplified in Figure 2. We added a concise explanatory paragraph at p. 10 describing the semantic relationships among the main entities. p.9 “The data model for the CoS is based on the knowledge based created for E-RIHS [7]. A selection of metadata fields was considered in order to respond to the requirements of the model centered on the service entity (Figure 2a-b), which acts as the main aggregation point and entry within the CoS. Each service is linked to one provider (i.e. an organization and a service managers) through explicit object properties that define responsibility for its implementation and delivery. Services are also associated with one or more platforms, representing the operational environments where they run, as well as with one or more tools and methods entities that describe the scientific approaches and technical capabilities they offer. These relationships ensure semantic consistency, interoperability, and machine-actionable service descriptions.”

**Request 4**: *How does this E-RIHS CoS fit into the overall E-RIHS system and the even broader European RIs? Are there use cases that users need to leverage E-RIHS CoS and other components of E-RIHS and/or European RIs to accomplish some objectives? Highlight its interoperability with other components of the E-RIHS system and/or the broader European RIs through those use cases.*

**Answer**: Thank you for requesting clarification on how the CoS fits within the wider E-RIHS system and European research infrastructures. To address this, we added a paragraph. We also clarify that all CoS data are harvestable and reusable by European research marketplaces, and that the Catalogue system is fully open source, enabling reuse and adaptation by other research infrastructures. Relevant references have been added. p. 11: “The new architecture and the use of open APIs increased interoperability with other tools developed within E-RIHS (DIGILAB) and with systems created in the context of other research infrastructures or projects at European and national level (e.g. EOSC, SSHOC, Resinfra, H2IOSC). As a result, all the data in the E-RIHS catalogue can be easily harvested and reused by the European marketplaces, expanding its accessibility. Furthermore, the catalogue system is fully released as open-source software, enabling its reuse and adaptation by other research infrastructures.”

**Request 5**: *“Scalability” is listed as one of the novelties of the E-RIHS CoS. But I can't seem to find information that elaborate what "scalability" means in this context, how scalability was achieved, and if there was any effort to quantify the scalability.*

**Answer**: Thank you for this request for clarification of the scalability concept. We added a dedicated paragraph and contextualised scalability within a catalogue for the heritage science community. pp.10-11 “A key aspect was the creation of an independent platform built on a scalable, modular architecture, in collaboration with Net7 ICT company 31, which is particularly important for a domain in constant change and for an RI in its initial operational phase. This design allows the platform to adapt and grow with the evolving needs of the RI and can be integrated with new features and emerging technologies. The E-RIHS CoS scalability resides in its core architectural and conceptual requirements since it is designed to accommodate growth in users, service records, and access requests without performance degradation, while supporting the progressive extension of functionalities through modular components and open APIs. At the same time, the data and semantic levels are handled through an extensible data model and controlled vocabularies that can evolve without compromising coherence or interoperability, facilitating the inclusion of new services originating from disciplines progressively incorporated into the scientific community, such as architecture, bioarchaeology, and related domains.”

**Request 6**: *What are the lessons learned from E-RIHS CoS that can potentially benefit the broader communities, beyond heritage science and European RIs?*

**Answer**: Thank you for this suggestion. We expanded the Conclusions by including some consideration describing how the CoS approach can impact other communities. p.16: “The development of the E-RIHS CoS demonstrates how a service-oriented digital platform can be used to structure and formalise the knowledge of a highly interdisciplinary research community through shared standards, common conceptual frameworks, controlled vocabularies, and FAIR-aligned data practices. Beyond the management of access to the scientific services, from proposal submission to delivery, the catalogue’s workflow acts as an enabling mechanism for knowledge co-creation, fostering collaboration among researchers with diverse expertise and objectives, highlighting how an interoperable user-centered framework can overcome disciplinary barriers and support sustainable and cross-domain research ecosystems for scientists and other specialists.”

**Request 7**: *The manuscript doesn't sufficiently describe how the AI-driven recommendation engine works. It says “Users’ engagement through input and feedback directly contributes to refining the system, allowing it to function as a dynamic, self-improving resource that supports decision-making, increases”. But it’s unclear how users’ input and feedback are used to refine the system.*

**Answer**: Thank you for this observation. We have elaborated more on the AI-driven recommendation system and the interaction with the users, as well as the strategies undertaken to improve the AI-system by including the contribution of the community. pp.13-14 “At the time this work was developed (2020-2025), a major innovation in development was the integration of a recommendation engine within the CoS, inspired by recommender systems used in e-commerce and media platforms. This AI driven component was designed to support users in identifying the most appropriate services based on their research needs, , having in mind that the community is broad and that not all users are experts in the scientific fields or equipment characteristics and methodological applications. The system is based on a Retrieval-Augmented Generation (RAG) architecture that integrates language models with a vector indexing infrastructure [9]. By generating high-dimensional embeddings and leveraging a vector database (Elasticsearch), the system performs similarity search operations to semantically map user queries to indexed content. The various strategies adopted – ranging from classical semantic similarity to multi-vector and multi-querying approaches – enable increased contextual granularity, improved robustness of information retrieval, and mitigation of model hallucination phenomena. The engine was designed to analyze a broad array of data, including equipment characteristics, methodological applications, past project outcomes, and user feedback, to generate personalized recommendations. Natural language queries are supported to increase accessibility and inclusiveness, especially for non-experienced users looking for the best solution for their research question. However, the AI-driven recommendation engine is not intended to replace the expertise of heritage scientists or the fundamental relationship between users and service managers. Rather, it serves as an initial orientation tool to guide users through the complex landscape of services and to build a more structured data foundation for this multifaceted sector. Data for training the algorithm are being collected through pilot applications distributed among the community and from E-RIHS service managers, with all processing conducted in full compliance with EU data protection regulations. Additionally, a dedicated working group identified representative research needs, compiling standardized controlled vocabularies and classification schemes to be used in service descriptions. The contribution of researchers has been critical in enhancing the robustness and reliability of the system. Questions automatically generated by the Large Language Model (LLM) underwent manual validation, transforming a synthetic dataset into a golden dataset of reference, essential for rigorous endpoint evaluation. This intervention significantly impacted not only the improvement of retrieval quality but also the establishment of a structured feedback framework, which is fundamental for calibrating embedding techniques and optimizing the entire RAG pipeline according to E-RIHS’s operational requirements. .... In a context marked by the rapid evolution of AI-driven technologies, targeted financial investments will be necessary to ensure future system updates and scalability.”

**Request 8**: *The "Current state of the CoS" section described how the training sessions and intermediate testing phases were conducted and "resulted in concrete enhancements across the platform". An elaboration of what feedback was collected during the training and testing and what enhancements were made based on these feedback would be helpful.*

**Answer**: Thank you for this suggestion. We have expanded the description of training sessions and UX testing results. p.14 “The new E-RIHS CoS platform marked a milestone in delivering integrated access to heritage science services. Its first version, including the dashboard system, was released in October 2024. This release followed an intensive phase of design, prototyping, testing, and iteration, culminating in the transition from development to operational deployment. From the earliest stages, service managers were engaged in the co-design of the system, contributing to data modelling, the selection of controlled vocabularies, and the definition of access procedures. To support the operational rollout, training sessions for service managers of the different platforms were conducted from October to December 2024, involving a total of 32 representatives from E-RIHS National Nodes to start inserting services ( Figure 5). They also served as validation environments, highlighting formal and functional requirements. Among the issues and changes identified in this phase can be listed the inclusion of a comment section from service managers to users following the feasibility evaluation to enhance the co-creation and collaboration between the user and the scientists. Also, the specialist involved identified the need to enable proposal revision and resubmission which required a revision in the functioning workflow. The sessions, which were recorded and distributed to reach a wider audience, also allowed to identify concepts that were not completely clear to the service managers and represented an opportunity to adjust, when necessary, and explain the data model at the foundation of the CoS.” p. 15 “To ensure methodological coherence in user-centred design, the IT company formally integrated a professional UX designer into the development team, supporting evidence-based design decisions and consistency across interfaces. UX testing was conducted in multiple iterative sessions involving different user profiles: (i) expert users already familiar with the IPERION HS catalogue, engaged to optimise task efficiency and reduce interaction latency; (ii) heritage science practitioners without prior exposure to the catalogue, involved to assess general usability and cognitive load; and (iii) non-specialist users (e.g. art historians) with no previous experience of the system, recruited to evaluate navigability, onboarding effectiveness, and critical friction points within the access workflow.”

**Request 9**: *An Excel spreadsheet (E-RIHS CoS_CNR INO_Dataset.xlsx) is listed as a dataset associated with this manuscript. But I couldn't find this anywhere this dataset was described in the manuscript.*

**Answer**: We are sorry if this was not completely clear. We have described the dataset in the Data Availability section. The dataset, derived from the operational database of the E-RIHS ERIC Catalogue of Services, is available via Zenodo as the CNR INO Dataset for the E-RIHS Catalogue of Services (DOI: 10.5281/zenodo.16532272).

# Reviewer report: Reviewer response for version 1

**DOI:** 10.21956/openreseurope.22502.r63539

**Published:** 2025-11-25

**Authors:** Katrina Exter

**Affiliations:** 1 Flanders Marine Institute (VLIZ), Oostende, Belgium

Nice arcticle, well written, very easy to read.

 The audience focus for the paper is others creating catalogues of services. This article provides well for that audience: much appreciated.

 The technical aspects are nicely introduced and summarised, and the GitHub space provides a lot of extra material for those who want to, to explore: much appreciated. (Tho a front-page README would be appreciated on that GH space)

 Two things are lacking

 - An explantion of the human side of the development. Often the biggest hurdle to creating anything new are the humans, not the IT. More detail on their experience on getting cooperation and good input from the service managers - what did and did not work - would be much appreciated. More detail on how the UX testing was designed, conducted, managed would also be appreciated, as user testing is crucial. 

 - AI: it is a little unclear how much of the AI-based  "recommendations" part of the CoS is already in place and how much is still being tested. If this is still in development, will another article on that be written later? As this is the big novel part of this CoS, there will be a lot of interest in know how it was done. What did AI bring to the table - especially as AI is so resource intensive, it should be justitifed why the AI approach is superior to any tested/investigated non-AI methods. What algorithms were used in the AI? Will this be open source, once completed (and if not, why)? 

 A suggestion: JSON-LD would be superior to JSON for your schemas (as listed in Table 1), adding much machine-understandablity (semantics).

If any, are all the source data and materials underlying the results available?

Yes

Is the work original in terms of material and argument?

Yes

Is the work clearly and cogently presented?

Yes

Is the argument persuasive and supported by evidence?

Yes

Does the research article contribute to the cultural, historical, social understanding of the field?

Partly

Does it sufficiently engage with relevant methodologies and secondary literature on the topic?

Yes

Reviewer Expertise:

Open science, data management

I confirm that I have read this submission and believe that I have an appropriate level of expertise to confirm that it is of an acceptable scientific standard, however I have significant reservations, as outlined above.

# Response

**Published:** 2026-01-21

**Authors:** Laura Benassi

Thank you for the positive and encouraging assessment of the article, as well as for recognising its relevance to communities involved in the design and implementation of catalogues of services. We also appreciate the comments regarding the clarity of the technical presentation and the availability of supplementary material through GitHub. We acknowledge the suggestion regarding the inclusion of a front-page README in the GitHub repository and will address this in a forthcoming update.

**Request 1**: An explanation of the human side of the development. Often the biggest hurdle to creating anything new are the humans, not the IT. More detail on their experience on getting cooperation and good input from the service managers - what did and did not work - would be much appreciated. 

**Answer**: Thank you for this suggestion. We fully agree that the human dimension represents a critical factor in the development of complex digital infrastructures and that this aspect deserves explicit discussion. We have expanded the manuscript to include the coordination effort, stakeholder engagement, and iterative collaboration with service managers and users. p.6: “Over the last years, a dedicated coordination group within the heritage science community has defined a shared conceptual framework for service description, metadata representation, and data structuring. This framework was iteratively discussed and refined in close cooperation with service managers (then referred to as “providers”) responsible for the delivery of individual services, ensuring both domain relevance and operational feasibility. The first online catalogue, conceived as a sort of e-commerce website, was released during the IPERION HS project in 2020. In coordination with the ARCHLAB, FIXLAB, and MOLAB platform leaders, services were clustered into high-level functional categories (e.g. spectroscopic analysis, spot analysis, etc.), leading to the formalisation of a coherent data model [5]. In the absence of a consolidated domain ontology or standardized controlled vocabularies for heritage science, service managers were invited to populate the model fields and contribute domain-specific terminology reflecting established practices within their scientific communities. The coordination group subsequently carried out an extensive, tricky process of data curation, harmonisation, and standardisation, validating semantic consistency and structural coherence across the catalogue.” p.7 “The redesign of the data model was made possible through regular meetings of a group dedicated to the catalogue’s development, during which different perspectives were discussed and iterative revisions were necessary. The users’ point of view was also considered and was included via targeted questionnaires distributed to experienced users and, more broadly, to the heritage science community through dedicated mailing lists.”

**Request 2:** *AI: it is a little unclear how much of the AI-based "recommendations" part of the CoS is already in place and how much is still being tested. If this is still in development, will another article on that be written later? As this is the big novel part of this CoS, there will be a lot of interest in knowing how it was done. What did AI bring to the table - especially as AI is so resource intensive, it should be justified why the AI approach is superior to any tested/investigated non-AI methods. What algorithms were used in the AI? Will this be open source, once completed (and if not, why)?*

**Answer:** Thank you for your consideration and suggestion. The adoption of AI within the E-RIHS Catalogue generated extensive discussion within the scientific community. On the one hand, it raised concerns regarding potential biases, such as the risk that the system might systematically recommend the same services. On the other hand, it also led to unrealistic expectations. Ultimately, the decision was to integrate a set of foundational AI functionalities to improve search performance and support the incremental development of the knowledge base required by the community. To clarify the role of AI, we integrated the text and included further discussion and information: p.13-14 “At the time this work was developed (2020-2025), a major innovation in development was the integration of a recommendation engine within the CoS, inspired by recommender systems used in e-commerce and media platforms. This AI driven component was designed to support users in identifying the most appropriate services based on their research needs, having in mind that the community is broad and that not all users are experts in the scientific fields or equipment characteristics and methodological applications. The system is based on a Retrieval-Augmented Generation (RAG) architecture that integrates language models with a vector indexing infrastructure [9]. By generating high-dimensional embeddings and leveraging a vector database (Elasticsearch), the system performs similarity search operations to semantically map user queries to indexed content. The various strategies adopted – ranging from classical semantic similarity to multi-vector and multi-querying approaches – enable increased contextual granularity, improved robustness of information retrieval, and mitigation of model hallucination phenomena. The engine was designed to analyse a broad array of data, including equipment characteristics, methodological applications, past project outcomes, and user feedback, to generate personalized recommendations. Natural language queries are supported to increase accessibility and inclusiveness, especially for non-experienced users looking for the best solution for their research question. However, the AI-driven recommendation engine is not intended to replace the expertise of heritage scientists or the fundamental relationship between users and service managers. Rather, it serves as an initial orientation tool to guide users through the complex landscape of services and to build a more structured data foundation for this multifaceted sector. Data for training the algorithm are being collected through pilot applications distributed among the community and from E-RIHS service managers, with all processing conducted in full compliance with EU data protection regulations. Additionally, a dedicated working group identified representative research needs, compiling standardized controlled vocabularies and classification schemes to be used in service descriptions. The contribution of researchers has been critical in enhancing the robustness and reliability of the system. Questions automatically generated by the Large Language Model (LLM) underwent manual validation, transforming a synthetic dataset into a golden dataset of reference, essential for rigorous endpoint evaluation. This intervention significantly impacted not only the improvement of retrieval quality but also the establishment of a structured feedback framework, which is fundamental for calibrating embedding techniques and optimizing the entire RAG pipeline according to E-RIHS’s operational requirements. ... In a context marked by the rapid evolution of AI-driven technologies, targeted financial investments will be necessary to ensure future system updates and scalability.”

**Request 3**: More detail on how testing was designed, conducted, managed would also be appreciated, as user testing is crucial.

**Answer**: Thank you for this suggestion. We have added and expanded several paragraphs describing the UX methodology and evaluation process (pp. 14–15), describing how providers were involved from the earliest stages in the co-design of the system, contributing to data modelling, controlled vocabulary selection, and access procedure definition. Additionally, we expanded the description of the iterative UX testing strategy. p.14: “From the earliest stages, service managers were engaged in the co-design of the system, contributing to data modelling, the selection of controlled vocabularies, and the definition of access procedures. To support the operational rollout, training sessions for service managers of the different platforms were conducted from October to December 2024, involving a total of 32 representatives from E-RIHS National Nodes to start inserting services (Figure 5). They also served as validation environments, highlighting formal and functional requirements. Among the issues and changes identified in this phase can be listed the inclusion of a comment section from service managers to users following the feasibility evaluation to enhance the co-creation and collaboration between the user and the scientists. Also, the specialist involved identified the need to enable proposal revision and resubmission which required a revision in the functioning workflow. The sessions, which were recorded and distributed to reach a wider audience, also allowed to identify concepts that were not completely clear to the service managers and represented an opportunity to adjust, when necessary, and explain the data model at the foundation of the CoS.” p.15: “To ensure methodological coherence in user-centred design, the IT company formally integrated a professional UX designer into the development team, supporting evidence-based design decisions and consistency across interfaces. UX testing was conducted in multiple iterative sessions involving different user profiles: (i) expert users already familiar with the IPERION HS catalogue, engaged to optimise task efficiency and reduce interaction latency; (ii) heritage science practitioners without prior exposure to the catalogue, involved to assess general usability and cognitive load; and (iii) non-specialist users with no previous experience of the system, recruited to evaluate navigability, onboarding effectiveness, and critical friction points within the access workflow.”

**Request 4:** JSON-LD would be superior to JSON for your schemas (as listed in Table 1), adding much machine-understandability (semantics).

**Answer: **Thank you for the suggestion. We agree that JSON-LD would significantly enhance machine-understandability by enabling explicit semantics and linked data interoperability. In the current implementation, standard JSON was adopted to ensure simplicity and broad adoption during the initial operational phase. However, the data model has been intentionally designed to support semantic extensions, and the adoption of JSON-LD, potentially through tools such as the *Dynamic Modeller* for improved formatting and JSON-LD handling, is being considered as part of future developments.
